# Asymptotic estimates of SARS-CoV-2 infection counts and their sensitivity to stochastic perturbation

**DOI:** 10.1063/5.0008834

**Published:** 2020-05-19

**Authors:** Davide Faranda, Isaac Pérez Castillo, Oliver Hulme, Aglaé Jezequel, Jeroen S. W. Lamb, Yuzuru Sato, Erica L. Thompson

**Affiliations:** 1 Laboratoire des Sciences du Climat et de l’Environnement, CEA Saclay l’Orme des Merisiers, UMR 8212 CEA-CNRS-UVSQ, Université Paris-Saclay and IPSL, 91191 Gif-sur-Yvette, France; 2 London Mathematical Laboratory, 8 Margravine Gardens, London W6 8RH, United Kingdom; 3 LMD/IPSL, Ecole Normale Superieure, PSL Research University, 75005 Paris, France; 4 Department of Quantum Physics and Photonics, Institute of Physics, UNAM, P.O. Box 20-364, 01000 Mexico City, Mexico; 5 Danish Research Centre for Magnetic Resonance, Centre for Functional and Diagnostic Imaging and Research, Copenhagen University Hospital Hvidovre, Kettegard Allé 30, 2650 Hvidovre, Denmark; 6 LMD/IPSL, ENS, PSL Université, École Polytechnique, Institut Polytechnique de Paris, Sorbonne Université, CNRS, 75005 Paris, France; 7 Ecole des Ponts, 77455 Marne-la-Vallé, France; 8 Department of Mathematics, Imperial College London, SW7 R2H London, United Kingdom; 9 RIES/Department of Mathematics, Hokkaido University, N20 W10, Kita-ku, Sapporo, Hokkaido 001-0020, Japan; 10 Centre for the Analysis of Time Series, London School of Economics and Political Science, Houghton Street, London WC2A 2AE, United Kingdom

## Abstract

Despite the importance of having robust estimates of the time-asymptotic total number of infections, early estimates of COVID-19 show enormous fluctuations. Using COVID-19 data from different countries, we show that predictions are extremely sensitive to the reporting protocol and crucially depend on the last available data point before the maximum number of daily infections is reached. We propose a physical explanation for this sensitivity, using a susceptible–exposed–infected–recovered model, where the parameters are stochastically perturbed to simulate the difficulty in detecting patients, different confinement measures taken by different countries, as well as changes in the virus characteristics. Our results suggest that there are physical and statistical reasons to assign low confidence to statistical and dynamical fits, despite their apparently good statistical scores. These considerations are general and can be applied to other epidemics.

COVID-19 is currently affecting over 180 countries worldwide and poses serious threats to public health as well as economic and social stability of many countries. Modeling and extrapolating in near real-time the evolution of COVID-19 epidemics is a scientific challenge, which requires a deep understanding of the non-linearities undermining the dynamics of the epidemics. Here, we show that real-time predictions of COVID-19 infections are extremely sensitive to errors in data collection and crucially depend on the last available data point. We test these ideas in both statistical (logistic) and dynamical (susceptible–exposed–infected–recovered) models that are currently used to forecast the evolution of the COVID-19 epidemic. Our goal is to show how uncertainties arising from both poor data quality and inadequate estimations of model parameters (incubation, infection, and recovery rates) propagate to long-term extrapolations of infection counts. We provide guidelines for reporting those uncertainties to the scientific community and the general public.

## I. INTRODUCTION

Severe acute respiratory syndrome coronavirus 2 (SARS-CoV-2), a zoonotic virus of the coronavirus family^1^ that provokes an infectious disease known as COVID-19, emerged from China at the end of 2019, affecting the Hubei province first and spreading quickly to all Chinese provinces.^2^ The failure of initial containment measures caused the virus to spread internationally, and on March 11, the World Health Organization (WHO) declared COVID-19 a pandemic.^3^ According to the WHO Situation Report-59 released on March 19,^4^ the number of countries affected by the pandemic was 176, with 209 839 confirmed infections and 8778 deaths. As this report also noticed: *the number of confirmed cases worldwide has exceeded 200 000. It took over 3 months to reach the first 100 000 confirmed cases and only 12 days to reach the next 100 000*, an astonishing development due to the highly contagious character of SARS-CoV-2.

SARS-CoV-2 causes a potentially life-threatening form of pneumonia in a non-negligible patient fraction.^5^ Enormous efforts to contain the virus and to not overwhelm intensive care facilities are currently being undertaken all over the world. Following the drop in infections observed in the Hubei province, restrictive confinement measures have been taken in many countries.^6^ Most of the time, those measures are taken on the basis of epidemic models, which are either dynamical or statistical models, and whose parameters are fitted with the available data.

COVID-19 data should be provided daily, following a request by the WHO. To date, the WHO guidelines require countries to report, at each day t, the total number of infected patients I(t) as well as the number of deaths D(t). Unfortunately, there is large variability in the way both I(t) and D(t) are counted. We provide some illustrative examples. On the one hand, Italy shows the highest fatality rate,

$$f=\sum_{t=1}^{\tau }D(t)/\sum_{t=1}^{\tau }I(t)≃0.07,$$ (1)

possibly because D(t) includes all deaths who had contracted SARS-CoV-2, independent of whether the virus was the actual cause of death. Moreover, in a recent interview,^7^ Italian biologist Bucci stated that D(t) can be underestimated because this does not include those patients who died at home without being tested. On the other hand, in Germany, the fatality rate is extremely low f≃0.002. Some query data methodology [e.g., a different method to determine D(t)], while others say high testing rates are giving a more accurate picture,^8^ although these hypotheses remain at the level of speculation.

Much uncertainty also exist in the count of I(t). While in the early stage of the epidemic, several countries tested asymptomatic individuals to track back the infection chain, recent policies to estimate I(t) have changed. Most of the western countries now test only patients displaying severe SARS-CoV-2 symptoms. In an effort to track all the chains of infections, South Korea has tested many asymptomatic people. This latter strategy has proven effective in supporting actions to reduce the rate of new infections. A recent study^9^ has estimated that an enormous number of total infections were undocumented (80% to 90%) and that those undetected infections were the source for 79% of documented cases in China.

The goal of this paper is to analyze the effect of those large uncertainties in real-time forecasting of the long-term behavior of the COVID-19 epidemic.^10^ As stated by Polonsky *et al.*,^11^ there is a need for defining robust methods to assess both the intrinsic errors inherent to fitting procedures as well as those introduced by poor data quality. Funk *et al.*^12^ give a concrete example of this applied to the Ebola epidemics in the Western Area region of Sierra Leone in 2014–2015. Classically, epidemiologists rely on Susceptible–Exposed–Infected–Recovered (SEIR) models.^13^ These models consist of ordinary differential equations where a population is divided into compartments, with the assumption that every individual in the same compartment has the same characteristics. In SEIR, the population is divided into susceptible, exposed, infected, and recovered individuals. Such models predict a sigmoid shape of the total number of infections $C(t)=\gamma \sum_{\tau =1}^{t}I(\tau )$. Using the available national data points I(t), one can obtain long-term estimates on the total of COVID-19 infections in each country. This paper focuses on the estimation of the sensitivity of these models to the last available data point before the inflection point of the I(t) curve is reached. We use SEIR models to show the possible origins of this sensitivity by perturbing the relevant parameters, often assumed deterministic, with a noise that mimics changes in the way the virus is spreading, e.g., as a result of application of confinement measures or the presence (rate/magnitude) of super-spreaders.^14^ The paper is organized as follows: in Sec. II, we discuss the various sources of data for COVID-19 and their shortcomings, and then we discuss in detail the SEIR model and its statistical modeling. In Sec. III, we discuss the results focusing on the statistical sensitivity of the modeling and apply them to the data from France, UK, and Italy. We finish, in Sec. V, with some remarks and point out some potentially beneficial policy guidelines.

## II. DATA AND MODELING

### A. Data

The data repository used in this paper for COVID-19 data is a Visual Dashboard operated by the Johns Hopkins University Center for Systems Science and Engineering (JHU CSSE). The data repository^15^ is also supported by ESRI Living Atlas Team and the Johns Hopkins University Applied Physics Lab (JHU APL). We used datasets of cases confirmed with a laboratory test, irrespective of clinical signs and symptoms.^3^ The data contain, as recognized by the public authorities that dispatched them, several inhomogeneities due to the different ways of testing patients with suspicious symptoms. As an example, Italy announced on February 26 that it relaxed testing criteria to the point that contacts linked to confirmed cases or recent travelers to outbreak areas would not be tested anymore, unless they show symptoms.^16^ Unlike Italy, South Korea (with a population of 51 million) has been testing 15 000–20 000 individuals per day since February 27 with the goal to minimize hospital pressure and stop the epidemic in the early stages.^17^ COVID-19 data also suffer from reporting problems due to the local management of health infrastructures. In Italy, healthcare is a regional task and every day data are collected at a regional level and transmitted to the Protezione Civile, who transfers the data to WHO. Many inconsistencies and delays have been documented in this transfer process.^18^ A similar situation occurs in Mexico, in which, for instance, private institutions, either hospitals or laboratories, do not possess the necessary national and international certifications given by the *Instituto de Diagnóstico y Referencia Epidemiológicos* (InDRE) and, therefore, their tests are not considered valid and must be redone by certified institutions,^19^ thus unnecessarily delaying of the release of accurate daily reports. COVID-19 data of Mexico were collected from the daily reports generated by Mexico’s *Secretaría de Salud*.^20^ Our goal is to account for these uncertainties in the modeling of COVID-19 data.

### B. An epidemiological Susceptible–Exposed–Infected–Recovered model

The Susceptible–Exposed–Infected–Recovered (SEIR) model^13^ is an epidemiological compartmental model where a total population N is divided into susceptible individuals S, exposed individuals E, infected individuals I, and the number R of people who have had the disease and are now either recovered or dead (and assumed not to be susceptible to reinfection). The model is constructed under the assumption that the total population N=S(t)+E(t)+I(t)+R(t) does not vary. This implies

0=dN/dt=dS/dt+dE/dt+dI/dt+dR/dt,∀t≥0. (2)

The model relies on some assumptions. First of all, susceptible individuals end up becoming infected and infected individuals can only recover or die. Individuals who are exposed (E) have had contact with an infected person but are not themselves infectious. Furthermore, those who have recovered or died are forever immune. It is also assumed that susceptibility is equal for all and that it is proportional to the product of I(t) and S(t) at a time t. These assumptions lead us to a set of four ordinary differential equations,

$$\begin{array}{rl} \frac{dS}{dt} & =-\lambda S(t)I(t), \end{array}$$ (3)

$$\begin{array}{rl} \frac{dE}{dt} & =\lambda S(t)I(t)-\alpha E(t), \end{array}$$ (4)

$$\begin{array}{rl} \frac{dI}{dt} & =\alpha E(t)-\gamma I(t), \end{array}$$ (5)

$$\begin{array}{rl} \frac{dR}{dt} & =\gamma I(t). \end{array}$$ (6)

Here, γ>0 represents the recovery/death rate or 1/γ the mean infection period, $\lambda =\lambda_{0}/S(0)>0$ is considered the contact or infection rate of the disease, and it is rescaled by the initial number of susceptible individuals S(0) and α is the inverse of the incubation period. These expressions satisfy (2) as required. Because data are reported only on a daily basis, we adopt the discrete SEIR model,

$$\begin{array}{rl} S(t+1) & =S(t)-\lambda S(t)I(t), \end{array}$$ (7)

$$\begin{array}{rl} E(t+1) & =(1-\alpha )E(t)+\lambda S(t)I(t), \end{array}$$ (8)

$$\begin{array}{rl} I(t+1) & =(1-\gamma )I(t)+\alpha E(t), \end{array}$$ (9)

$$\begin{array}{rl} R(t+1) & =R(t)+\gamma I(t). \end{array}$$ (10)

**FIG. 1.**
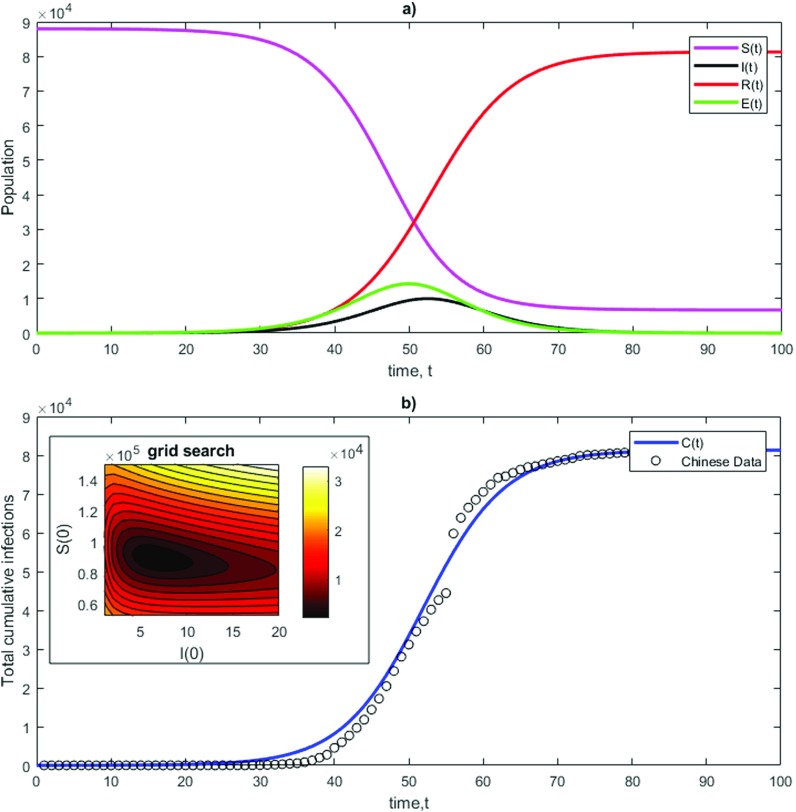
Example of a Susceptible–Exposed–Infected–Recovered (SEIR) model of COVID-19 [Eqs. (7)–(10)] with λ=1/S(0), α=0.27, γ=0.37. Initial conditions are set to I(0)=6, S(0)=88000, E(0)=R(0)=0. (a) Time evolution for the variables of the system; (b) time evolution for the total number of infections C(t) against the Chinese data with t=1 corresponding to December 19, 2019. The inset shows the outcome of the grid search procedure, where the root mean square error between the Chinese data and the modeled C(t) is minimized.

This model is obtained rewriting the ordinary differential equations (3)–(6) with an Euler scheme and fixing dt=1 day. An important derived quantity of the model is $R_{0}=\lambda_{0}/\gamma$, the average reproduction number of the virus in a population. This quantity represents the number of cases, on average, an infected person will cause during their infectious period. For COVID-19 in Wuhan in January 2020, $R_{0}=2.68$ with 95% CrI 2.47−2.86 according to an estimate performed with the Wuhan data.^21^ Dynamical modeling of COVID-19 epidemic has been proposed in Ref. 22. In that study, the authors used a Susceptible–Exposed–Infected–Recovered model with delays and performed a sensitivity study on the parameters. Fixing λ≃1 as in Ref. 22 and γ=0.37 to recover the value of $R_{0}$ found in Ref. 21 (assuming that the behavioral elements of viral transmission are consistent in other populations), we are left with the choice of α. The range for incubation period of SARS-CoV-2 has been determined in Ref. 23 between 2 and 11 days. As a comparison, this range is estimated to be between 2 and 5 days for human coronavirus and between 2 and 10 days for severe acute respiratory syndrome (SARS) coronavirus.^24^ Here, we set α=0.27 (corresponding to an incubation period between 3 and 4 days). Using a grid search procedure where both I(0) and S(0) are tested and using the root mean square error between Chinese data and the modeled C(t), we obtain the best fit when initial conditions are S(0)=88000, I(0)=6, E(0)=R(0)=0. The fit against the Chinese data is reported in Fig. 1, and the grid search optimization is shown in the inset. The best-fit yields a root mean square error of ∼2500, which represents about the 20% of the peak value of I(t) in the Chinese data. First of all, we note that, despite its simplicity, the model shows qualitatively similar behavior to the published data. Note that there is a discontinuity in the dataset, which is due to a change in the way infections were counted, introduced on February 12, 2020.^25^

This model also has evident deficiencies in representing the COVID-19 infections. First of all, the total population N, which provides the best fit for the Chinese data, is orders of magnitude lower than that of China or the Hubei province. Indeed, a major problem in the estimation of the SEIR model for COVID-19 is almost the total absence of infection counts for asymptomatic patients. In Ref. 26, posterior model estimates of percentage of total population infected (prevalence), as of March 28, 2020, have been performed for European countries that yield a ratio between C(t) and total population of the same order of magnitude of the Chinese Hubei province. That study revealed a COVID-19 prevalence of 15% (CrI [3.7%–41%]) for Spain and 9.8% (CrI [3.2%–26%]) for Italy. Furthermore, the population under consideration does not consist of a group of about the same age and general health level, and the group members do not mix homogeneously. The model does not have any spatial component, nor does it predict the influences of policy and behavioral responses to the progress of the pandemic. Finally, the fit is obtained with a constant value of $R_{0}$, although confinement measures have been introduced, possibly leading to a reduction in $\lambda_{0}$ and, therefore, in $R_{0}$. More complex models introducing further parameters would likely lead to overfitting and overconfident predictions due to the limited volume of data currently available. No model will be sufficient to predict the outcome of this pandemic: the outcome depends on our response. Models are presented here with the aim of generating some insight into the overall behavior and the risks entailed by inaction.

### C. Statistical modeling

When insight is limited and compartmental models are not suited, phenomenological statistical models provide a starting point for estimation of key transmission parameters, such as the reproduction number, and forecasts of epidemic impact.^27^ One of the simplest ways to model the epidemics is to observe that the function C(t) is a sigmoid function and perform a statistical fit of the data to extrapolate the long-term behavior of the epidemics.^28,29^ Among all the possible sigmoid functions, two have proven useful in fitting epidemic growth: the generalized logistic distribution^30^ and the generalized Gompertz distribution.^31^ A complete overview of sigmoid functions is presented in Ref. 32, although applied to in a different context. Since our considerations are independent of the sigmoid function used, we will present results for the generalized logistic model only. The model reads

**FIG. 2.**
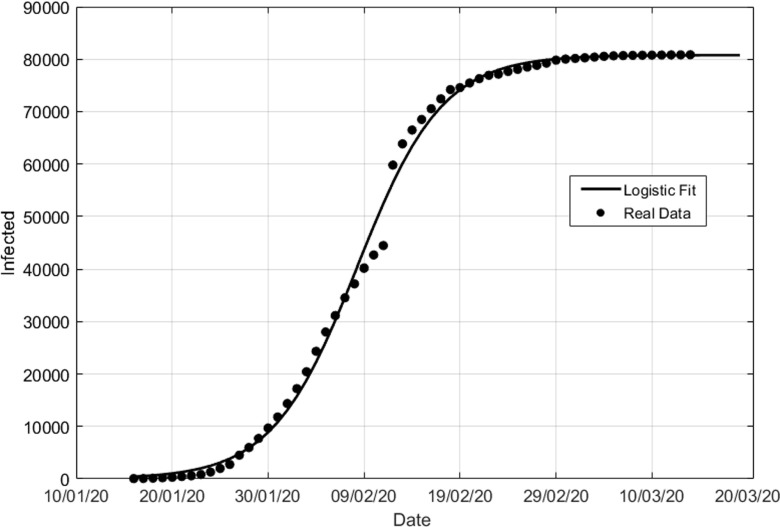
Logistic [Eq. (11)] fit of the Chinese number of infections C(t). The best-fit parameters are a=80800±400, b=0.225±0.005, c=190±25.

C(t)=a/(1+b⋅exp⁡(−c⋅t)), (11)

where a, b, and c are parameters of the model. They are linked in a non-explicit way to the solution of the SEIR model. A fit to the Chinese data is presented in Fig. 2. Logistic fits are performed with the MATLAB Nonlinear least-squares solver constraining objective function with gradient. At first sight, one can be tempted to use $R^{2}≃0.997$ as a quality indicator of the fit. However, we stress that $R^{2}$ is not an appropriate measure for nonlinear regression models: given the smoothness of data, there will be lots of models (e.g., low-order polynomial), which could fit well (get a very good $R^{2}$) but would not make credible predictions.^33^ These data are, however, collected at a mature stage of the epidemic and as such the characteristics of the logistic fit to these data can be assigned with greater confidence. In Sec. III, we will discuss the performance of the statistical model in the early stage of the epidemics, where the logistic function can be used to extrapolate the behavior of C(t).

## III. RESULTS: STATISTICAL AND DYNAMICAL MODELING OF EARLY STAGES OF THE EPIDEMICS

### A. Statistical sensitivity

**FIG. 3.**
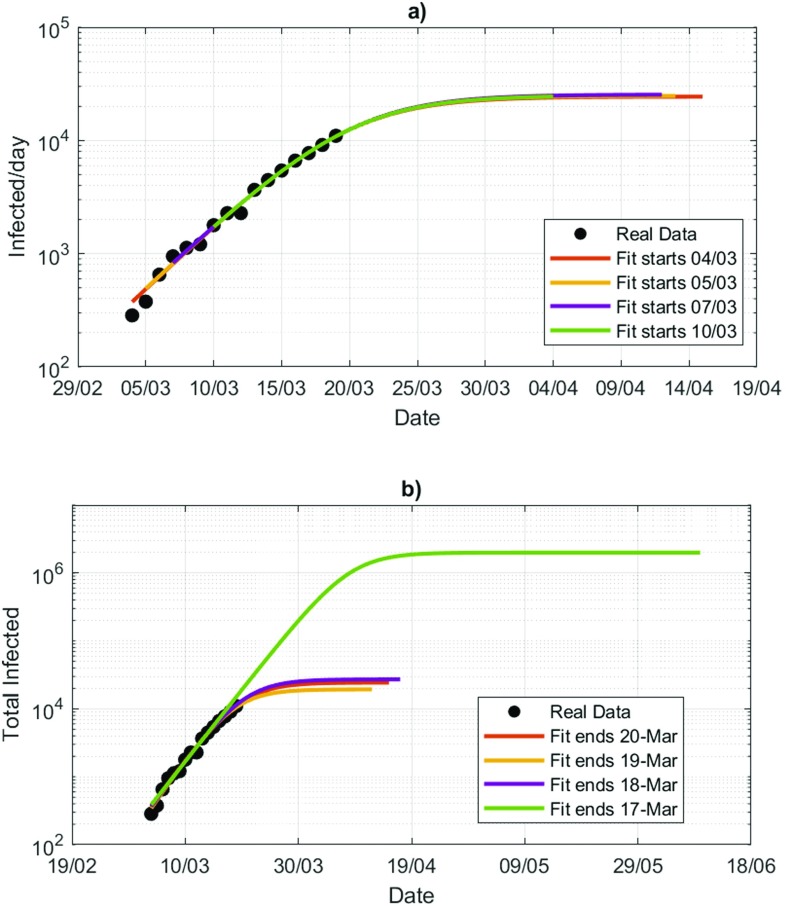
Logistic distribution fits for the early stages of the epidemic in France. (a) Logistic fits with data starting from different dates and ending on March 20. (b) Logistic fits ending on different dates but starting from March 4.

We begin by showing the sensitivity of the logistic extrapolations in the early stage of the epidemics by looking at the French data from March 4 to March 20. France has previously recorded sporadic cases of SARS-CoV-2 infections, but the exponential growth phase started at the beginning of March 2020. To show the high sensitivity to the last point of the datasets, we first perform a logistic fit with data starting from different dates and ending on March 20 [Fig. 3(a)] and then do the reverse experiment by fitting data starting on March 4 but ending at different dates [Fig. 3(b)]. This procedure is known as leave-one-out cross-validation, which has already been used in epidemiological models,^34^ although other studies have suggested that cross-validation is biased toward more complex models.^35^ Our goal is to use cross-validation not as a way to perform model selection but rather to assess the uncertainty in the estimation of the logistic fit to COVID-19 data. The results show that fits are more stable by removing days from the beginning of the outbreak than from the most recent past, therefore showing a time-asymmetry in the cross-validation procedure. Again, we stress the inadequacy of the $R^{2}$ metric as it yields values above $R^{2}>0.99$ for all cases considered in Fig. 3. The analysis suggests that, if a large error is presented in the last data point, the extrapolation has less predictive adequacy. This implies very narrow estimates of confidence intervals for C(t): for each fit, confidence intervals are as small as the thickness of the line used in the plots in Fig. 3. This prevents a correct evaluation of the confidence interval, which is critical to assess the uncertainties around the future evolution of the epidemics and to build relevant policies to address the worst case scenario.

**FIG. 4.**
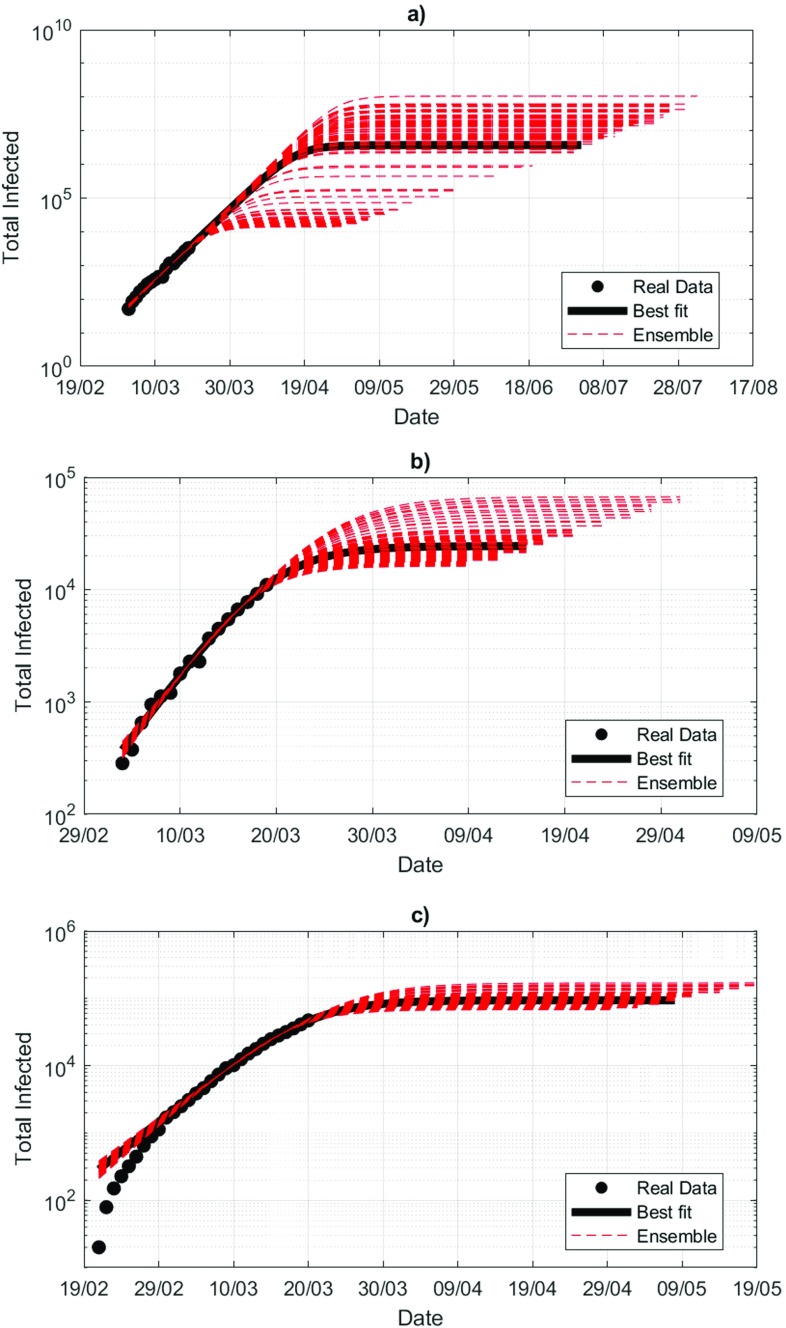
Logistic distribution obtained substituting the last data point with a random number $\xi (t^{*})$ drawn from a uniform distribution with mean $C(t^{*})$ and standard deviation $0.2C(t^{*})$ for the UK (a), France (b), and Italy (c).

To further test this concept, we now assume that we are uncertain about the magnitude of the last data point $C(t^{*})$. To simulate this uncertainty, we replace it with a random number $\xi (t^{*})$ drawn from a discrete uniform distribution with mean $C(t^{*})$ and standard deviation $0.2C(t^{*})$. The factor 0.2 has been chosen coherently with the root mean square error analysis performed during the grid search in Sec. II. We, therefore, construct an ensemble of 100 possible trajectories under this generative process. Results are presented in Fig. 4 for UK (a), France (b), and Italy (c). To date, Italy is at a more mature stage of the epidemic, while France and UK face an earlier stage. This is reflected in the spread of the ensemble: for the UK, forecasting the epidemic with a logistic fit is not informative of the course of the epidemic: the ensemble spread just suggests that the current phase is an exponential growth and at best it can inform that worst case scenarios should be considered at this point. The ensemble spread reduces when the epidemics is at a more mature stage (Italy). Indeed, if we set b=1 and we start the fit from time $t_{0}$, then the logistic distribution is written as

$$C(t)=a/(1+\exp(-c(t-t_{0}))).$$

In the early growth phase, $\exp(-c(t-t_{0}))\gg 1$, so

$$C(t)∼a\exp(c(t-t_{0}))=a\exp(-c\cdot t_{0})\exp(c\cdot t)=A\exp(c\cdot t).$$

Even though we can fit A and b to data, recalling that $A=a\exp(-c\cdot t_{0})$, we have that an error in c propagates exponentially into an error in a, the upper asymptote that determines the final count of the epidemics. The same sensitivity test for the middle, and the first data point has shown very little variability of the logistic fits.

### B. Dynamical sensitivity in a stochastic SEIR model

**FIG. 5.**
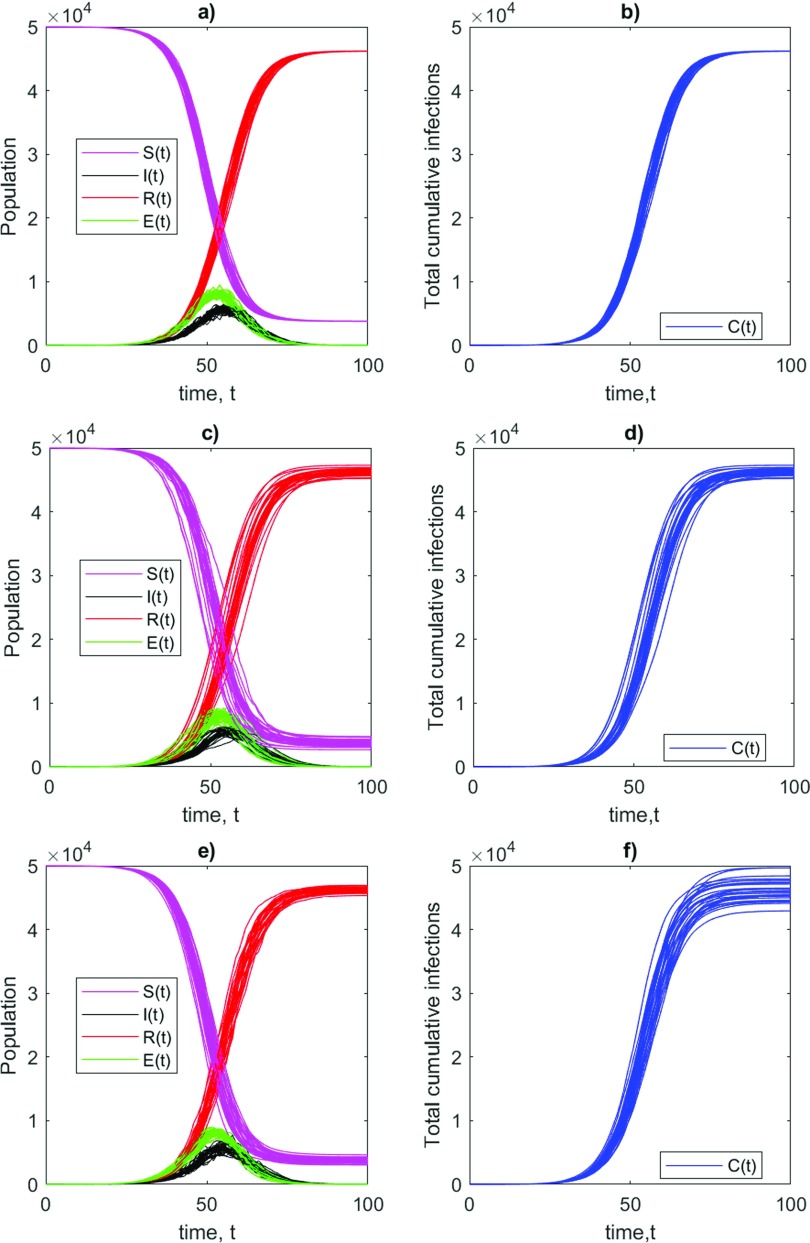
Example of 30 trajectories of dynamics of stochastic Susceptible–Exposed–Infected–Recovered (SEIR) model for COVID-19, obtained replacing alternatively α [(a) and (b)], λ [(c) and (d)], and γ [(e) and (f)] with the stochastic process in Eq. (12). Dynamics are integrated with a fixed initial conditions I(0)=2, S(0)=50000, E(0)=R(0)=0. (a), (c), and (e) Time evolution for the variables of the system; (b), (d), and (f) Time evolution for the total number of infections C(t).

Another way to understand the sensitivity in epidemics is to release the assumption that incubation period α, infection rate λ, and recovery rate γ are constant through the epidemics.^36^ Intrinsically, they can vary because of the presence of individuals with an extremely high transmission rate known as super-spreaders^14^ or due to the release or the application of confinement measures or changes in the SARS-CoV-2 characteristics. They can also display spurious variations due to the way data are reported or collected for the problems specified above. We explore all these possibilities by considering α, λ, and γ as time-varying processes. The idea of using stochastic models to represent epidemics is not new to the literature.^37–39^ In the modeling of COVID-19 infections, the stochastic approach can be further justified by the evidence that $R_{0}=\lambda /\gamma$ displays spatial and temporal variability.^11^ For example, Wu *et al.*^21^ show fluctuations of $R_{0}$ in different Chinese regions. These differences are due to changes in the duration of contagiousness, likelihood of infection per contact and the contact rate,^40^ which depends on demographic spatial variability.^41^ There is, however, little consensus on which variables or parameters should be perturbed in order to get a realistic behavior. Our goal here is different than obtaining the best possible forecasts of the epidemics as we want to understand which parameter causes a large sensitivity in the final C(t) counts. Let us begin, by alternatively replacing in Eqs. (7)–(10) one of the constant parameters κ∈{α,λ,γ} with a stochastic process

$$\kappa (t)=|\kappa_{0}+\sigma \cdot \xi (t)|,$$ (12)

where σ is the intensity of the perturbation and ξ(t) a random variable drawn from a normal distribution N(0,1) at each time. The absolute value avoids negative values of κ(t). The purpose of Eq. (12) is to introduce instantaneous discrete jumps in the values of the daily parameters. This discrete process, used in Ref. 42, is more appropriate than a continuous one (see, e.g., Ref. 43) when observations are affected by large detection errors, as in the present case. Figure 5 shows an example of 30 realizations of a stochastic SEIR COVID-19 model, obtained by replacing alternately α [5(a) and 5(b)], λ [5(c) and 5(d)], and γ [5(e) and 5(f)] with the stochastic process in Eq. (12) and using $\sigma =0.2\kappa_{0}$ to get fluctuations of the order of 20% of each parameter values, in analogy with the statistical sensitivity studies performed Sec. III A. The sensitivity clearly depends on the perturbed parameter: a perturbation on α mostly implies a different timing of the epidemics while the final cumulative number of infections C(t) remains unchanged. Perturbations on λ and γ affect the final C(t) in a deeper way, leading to a total variation in the number of cases of the order of 20%. Indeed, by changing λ and γ, we also modify the basic reproduction number $R_{0}$. The idea of having a time-varying reproduction number has been already exploited in Ref. 44, although the authors have directly modeled the dynamics of a dynamic reproduction number R(t) without introducing a SEIR model.

**FIG. 6.**
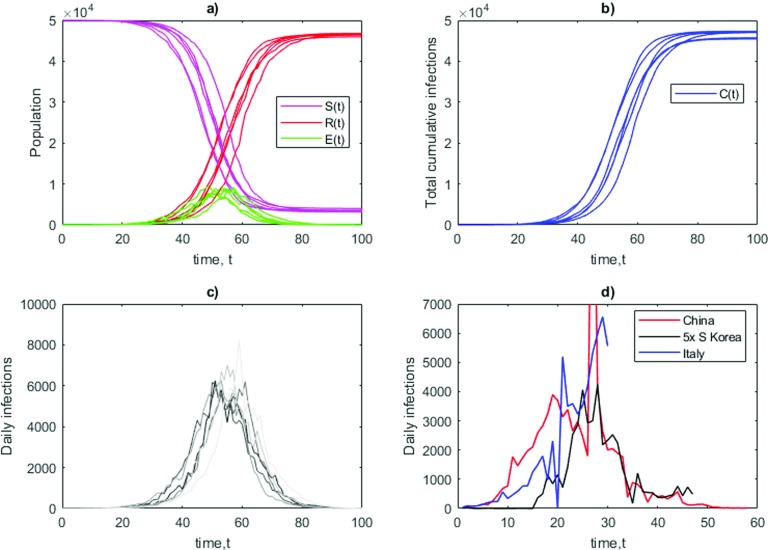
Example of six trajectories of dynamics of stochastic Susceptible–Exposed–Infected–Recovered (SEIR) model for COVID-19, obtained replacing all parameters α, λ and γ with an independent stochastic process as in Eq. (12). Dynamics are integrated with a fixed initial conditions I(0)=2, S(0)=50000, E(0)=R(0)=0. (a) Time evolution for the variables of the system. (b) Time evolution for the total number of infections C(t). (c) Time evolution for the daily infections. (d) Comparison with daily infections in China (red, starting December 19, 2019), South Korea (black, starting January 30, 2020), and Italy (blue, starting February 20, 2020).

As a further step, we add noise simultaneously to all parameters of the SEIR model via Eq. (12). Six realizations of the model are shown in Fig. 6. Figures 6(a) and 6(b) show the evolution of S(t), R(t),E(t), and C(t). We have separated the time evolution of I(t) in Fig. 6(c) to compare it with that of COVID-19 data for China, South Korea, and Italy [Fig. 6(d)]. Despite having a quasi-smooth behavior of C(t), we observe a highly non-smoothness of I(t), which is reflected by the data. The sensitivity of the model is higher when I(t) is large because γ and λ directly act on I(t). Therefore, when approaching the maximum of I(t) (t∼50 days), small changes in the parameters can greatly affect the final total count of infections C(t). This implies that mitigation strategies based on the reduction of λ by self-isolation, social distancing, are way more effective if imposed at the early stage of the epidemics because they allow to suppress the fluctuations in $R_{0}$ that can lead to spikes of I(t) and trigger a cascade infection process.

## IV. GUIDELINES FOR REAL-TIME EXTRAPOLATION OF EPIDEMIOLOGICAL DATA

Real-time forecasts of COVID-19 epidemics are crucial to plan the duration of confinement measures and to define the needs for healthcare facilities. Due to the intrinsically non-linear nature of the underlying dynamics, extrapolations of total infection counts depend not only on the quality of data but also on the stage of the epidemics. This prevents from performing successful long-term extrapolations of the infection counts with statistical models. On the basis of the results obtained, we can, however, define a few guidelines for the real-time dynamical and statistical models of the epidemics.

*Dynamical modeling*: Without having reliable estimates of the prevalence of the epidemics including asymptomatic patients, one would not expect quantitative forecasts from dynamical models such as SEIR to be correct, not even within an order of magnitude. A dynamical model only tells us something about the basic structure, or shape, of the epidemic. It is robust, for instance, that there is an exponential regime and that the outcome of the epidemics is very sensitive to variations in the parameters during the exponential phase. In order to use dynamical models, one should first perform a grid search for the deterministic SEIR model and obtain the best set of parameters. From the root mean square errors, one can infer the typical distance from model to data and use that value to set the level of noise in the parameters. Then, by running a stochastic SEIR model, an uncertainty range for the prediction can be obtained. If confinement measures are introduced, the estimate of $R_{0}$ should account for a reduction of λ, the contact rate, e.g., via the use of mobility data.

*Statistical modeling*: A simple cross-validation can follow both the approaches described in this paper: (i) exclude the last data points and check the stability of the estimates and (ii) add noise to the last data point and obtain an ensemble of estimates. Another approach could be based on evaluating every day each model on the performance in predicting the new data point, and then used again with the new data point for an updated estimate.

## V. DISCUSSION

In this work, we have discussed the statistical and dynamical sensitivity of asymptotic estimates of COVID-19 infections when performed at the early stages of the epidemics. First of all, we noted that SEIR model, with λ, γ, and α inferred from clinical studies, can fit Chinese data with a value of N≃88000 that is very different from that of the Chinese, Hubei, or Wuhan populations. This enormous discrepancy can be due both to a large underestimation in the prevalence or to the effectiveness of confinement measures which results in a smaller exposed population. This estimate should be taken as a first caveat in fitting a SEIR model to infer COVID-19 epidemics evolution in other countries as results may be largely under/overestimated.^11^

Then, we have shown that statistical fits often used to extrapolate the long-term behavior of the epidemics are greatly affected by the magnitude of the last data point, despite values of $R^{2}$ close to one, leading to unrealistic or overconfident estimates of confidence intervals on the forecast of the total number of infections.^45,46^ In the early stage of the epidemics, we have shown that knowing the last data point with a relative 20% error can lead to a final extrapolation of infections with an error of several orders of magnitude. In order to improve the estimates of statistical models, one should replace $R^{2}$ estimates by a formal comparison of model-alternatives using information criteria (e.g., AIC or BIC) or a log-likelihood approach with a leave-one-out cross-validation procedure.

Finally, we have investigated whether this statistical sensitivity can be dynamically reproduced with a SEIR model, where parameters are considered stochastic processes [Eq. (12)]. We have found that the stochastic dynamics are more sensitive to γ and λ. Perturbations on these parameters are proportional to the number of infected patients I(t) and are, therefore, important in the growth phase of the epidemics. Actual data display fluctuations even larger than those simulated in the stochastic models, suggesting that instead of assuming observational Gaussian noise on the parameters, jump processes (e.g., Levy noise) may be more appropriate.^47^ Furthermore, we noticed that large fluctuations in the number of detected infections are also due to changes in the testing protocols and availability of tests. All these inconsistencies prevent the possibility of performing meaningful asymptotic statistical or dynamical modeling for COVID-19 or comparing results among different countries. This may be even more problematic in less developed countries, which are just beginning to register cases.^48–50^

Our study suggests that dynamical and statistical modeling should focus on limited stages of the epidemics and restrict the analysis to specific regions, thus accounting for large uncertainties, as done in Ref. 51. Modeling approaches should take into account both statistical uncertainties as well as expert knowledge in a sort of Bayesian framework that allows to guide the choice of prior probabilities.^10^ In the interest of preserving the public health of as many individuals as possible, once modeled the uncertainty in the data, the worst case scenarios should always be taken into account very seriously as a guideline to enforce strict confinement measures.

## Data Availability

Raw data that support the findings of this study are openly available in Johns Hopkins University Center for Systems Science at https://systems.jhu.edu/research/public-health/ncov/. Derived data supporting the findings of this study are available from the corresponding author upon reasonable request.
